# Korean Proficiency Tests for Pesticide Residues in Rice: Comparison of Various Proficiency Testing Evaluation Methods and Identification of Critical Factors for Multiresidue Analysis

**DOI:** 10.3390/foods12102085

**Published:** 2023-05-22

**Authors:** Hyosub Lee, Gunhee Jung, Juhyeon Min, Hyanghee Kim, Wontae Jeong, Taekkyum Kim

**Affiliations:** 1 National Institute of Agricultural Sciences, 166 Nongsaengmyeong-ro, Iseo-myeon, Wanju-gun 55365, Republic of Koreayamche79@korea.kr (H.K.); shewaspretty@korea.kr (W.J.);; 2 Department of Food Science and Technology, Kyungpook National University, 25, Sangyeok-ro 14-gil, Buk-gu, Daegu 41536, Republic of Korea

**Keywords:** pesticide, proficiency test, z-score, SWZ, SZ2

## Abstract

Establishing pesticide safety management for agricultural products necessitates accurate pesticide analysis at analytical laboratories. Proficiency testing is regarded an effective method for quality control. Herein, proficiency tests were carried out for residual pesticide analysis in laboratories. All samples satisfied the homogeneity and stability criteria of the ISO 13528 standard. The obtained results were analyzed using the ISO 17043 z-score evaluation. Both individual pesticide and multiresidue proficiency evaluations were performed, and the proportion of z-scores within the ±2 range (“Satisfactory” rating) obtained for seven pesticides ranged 79–97%. Of the laboratories, 83% were classified as Category A using the category A/B method, and these also received AAA ratings in the triple-A evaluations. Furthermore, 66–74% of the laboratories were rated “Good” via five evaluation methods based on their z-scores. The sum of weighted z-scores and scaled sum of squared z-scores were considered as the most suitable evaluation techniques, as they compensated for the drawbacks of good results and corrected the poor results. To identify the main factors affecting laboratory analysis, the experience of the analyst, sample weight, calibration curve preparation method, and cleanup status were considered. A dispersive solid phase extraction cleanup significantly improved the results (*p* < 0.01).

## 1. Introduction

To ensure the safety of agricultural products, detecting that pesticide concentrations are below their maximum residue limits (MRLs) is essential. Pesticide residue analysis in agricultural products prohibits non-compliant foods from entering the market and ensures that compliant products do not receive non-compliant judgments [1,2]. However, a high level of expertise is required to accurately detect pesticides at a level of 0.01 mg/kg while removing various sample impurities. Thus, achieving a proper balance between the safety and cost-effectiveness of the analytical procedure is critical [3]. Recently, advanced analytical instruments, such as liquid chromatography–tandem mass spectrometry (LC–MS/MS) and gas chromatography–tandem mass spectrometry (GC–MS/MS), have been successfully used in residual pesticide analysis, and have thus enabled a rapid and convenient multiresidue analysis [4,5]. Nonetheless, simplification of the analytical process may result in analytical errors including false positives and false negatives owing to matrix effects, which depend on the experience of the analyst and laboratory quality control [6,7,8,9].

Since 2019, South Korea has implemented the Positive List System, which adopted 0.01 mg/kg as a standard for all pesticides without established MRLs in agricultural products. In 2021, the number of target analytes for managing agricultural products at the pre-harvest stage increased from 320 to 464, thus placing a greater burden on analysts. Consequently, the precise and accurate detection of these compounds is essential to minimize damage to farmers and consumers.

The role of local agricultural technology centers in Korea, which are closely connected to agricultural fields, is the inspection of residual pesticides in agricultural products before cultivation has expanded. As a result, the number of new analysts joining the field has increased. Quality control is essential for bridging the proficiency gap between new and experienced analysts and for increasing their overall proficiency level to obtain reliable results.

Various quality control methods can be employed to improve the reliability of an analytical procedure. First, developing standard operating procedures can standardize various stages of the analytical process, such as sample collection and preparation and instrument calibration [10]. The analytical methods used in routine analyses should be periodically inspected to verify their linearity, limits of quantification, recovery rates, and relative standard deviations [11,12,13]. The instruments used should be calibrated and maintained to check for contamination, injection volume, limit of detection, and analytical conditions [14]. The use of certified reference materials is required to verify the accuracy and traceability of the analytical process [15,16]. Although these steps are crucial for improving analytical accuracy, they are classified as internal quality management. Meanwhile, external quality management such as proficiency testing is required to maintain stable results at the national level and enhance their reliability [17,18].

Proficiency testing is a method for evaluating the analytical performance and accuracy of laboratories via interlaboratory comparisons [17]. In the field of residual pesticide analysis, proficiency testing is conducted on suitable samples that have undergone homogeneity and stability tests according to the ISO 13528 standard by adding arbitrary amounts of pesticide residues to agricultural products. The obtained results are then evaluated by calculating their z-scores according to the ISO 17043 standard [19]. The advantages of proficiency evaluation include the improvement of laboratory proficiency via quality control, method validation, and sharing best practices among different laboratories, thus ultimately increasing consumer confidence in agricultural products [5,6,20].

Residual pesticide analysis often involves multiresidue analysis, and proficiency testing evaluates the qualitative and quantitative analyses of various pesticides. Although a single-residue z-score evaluation method based on ISO 17043 can be used to assess individual compounds, it has several limitations in realistically evaluating laboratory proficiency. Although the Category A/B method, which is currently used in European Union proficiency tests (EUPTs), can evaluate laboratory proficiency, it does not consider the z-score of each compound. Therefore, Valverde et al. [21] proposed the triple-A evaluation method which adds false-positive factors to the category method that originally evaluated X (analytical range) and Y (z-score suitability proportion). Various techniques have been suggested for evaluating the capabilities of multiresidue analysis for all pesticides analyzed in proficiency tests [22] such as the rescaled sum of z-scores (RSZ) and sum of squared z-scores (SSZ) methods. However, the accuracy of these methods is limited because the z-scores of good evaluations can compensate for the poor results [22,23,24]. To overcome these limitations, the relative laboratory performance (RLP) method, which divides the number of pesticides in the existing SSZ data, has been proposed. In this method, the performance is classified into four groups: good, satisfactory, questionable, and unsatisfactory [25,26,27,28]. Medina-Pastor et al. [22] proposed the sum of weighted z-scores (SWZ) and scaled sum of squared z-scores (SZ2) methods based on the existing SSZ and RSZ methods, respectively. These two techniques reduced the correction effect of the good z-scores for poor results by adjusting their weights. Consequently, all of the aforementioned methods must be systematically compared to identify which is most suitable.

One of the critical factors strongly affecting pesticide analysis results is the preprocessing step [28]. Therefore, reviewing the currently applied routine analytical procedures and developing accurate analytical methods can considerably improve the quality of analysis.

In this study, we prepared proficiency test samples based on the ISO 13528 and 17043 standards and implemented a proficiency program targeting residual analysis laboratories. The results submitted by the participating laboratories were then employed to perform a comparative study to determine the most appropriate method among the various techniques to evaluate individual pesticides and laboratory proficiency. The critical factors affecting the accuracy of the analysis process conducted in a laboratory were finally identified.

## 2. Materials and Methods

### 2.1. Overview of the Proficiency Test Program in Korea

The proficiency test involved the preparation of brown rice samples for multiresidue analysis, which were subsequently distributed to the analytical institutions. The proficiency test was conducted in accordance with the ISO 17043 standard and EUPT general protocols. In total, 146 target compounds were evaluated, and seven types of pesticides were added for qualitative and quantitative analyses. The institutions participating in the proficiency test were safety analysis laboratories of agricultural technology centers in Korea which primarily conduct multiresidue pesticide analyses of agricultural products. The testing was performed between September and December 2021. The samples were delivered over a period of 3 day, after which the participating laboratories analyzed them within 30 d. The obtained results were evaluated by computing the z-scores.

### 2.2. Preparation of Multiresidue Proficiency Test Samples

Brown rice, the most commonly used agricultural product in Korea, was selected for proficiency testing. Pesticide-free brown rice and rice flour produced from this rice were purchased from a supermarket. Seven pesticides (chlorantraniliprole, difenoconazole, fenoxanil, ferimzone, hexaconazole, imidacloprid, and isoprothiolane) were selected as additives based on their high frequency of detection while monitoring residual pesticides in agricultural products distributed by the National Agricultural Products Quality Management Service (2017–2020). Proficiency test samples were prepared by adding 0.05–0.25 mg/kg of the seven pesticide standards dissolved in acetonitrile to ground brown rice (0.8 kg) based on the final sample weight (8.0 kg). After adding a standard solution to the brown rice (0.8 kg), the organic solvent was evaporated for 1 h, and the resulting mixture vigorously shaken for 1 min and stabilized for 24 h at 4 °C. Rice flour (7.2 kg) and pesticide-added rice flour (0.8 kg) were then homogenized in an industrial mixer (DKM-210 S-3, Daekwang, Republic Korea) for 8 h at 18 °C. To prepare untreated proficiency test samples, the same process was performed using pesticide-free brown rice. After homogenization, the samples were packed inside thermal bags with ice packs and delivered to the participating laboratories.

### 2.3. Homogeneity and Stability Tests of Proficiency Test Samples

Proficiency test samples must ensure the homogeneity and storage stability of pesticides throughout the testing period. For homogeneity and stability testing, ten samples were randomly selected after homogenization. The obtained results were analyzed twice by taking 5 g of each sample, and the pretreatment method used was EN QuEChERS. The standard deviations of the sample results were calculated using Equation (1) according to the ISO 13528 standard. Cochran’s test was conducted to check for outliers in the analysis results before evaluating the homogeneity of the sample. If the value obtained by dividing the square of the difference between the analysis values within the sample by the total sum of difference in squares within samples (D^2^) was less than the threshold value of 0.602 for ten samples, the absence of outliers was confirmed. Homogeneity was evaluated by calculating the allowable standard deviation threshold for the analyzed samples based on the F-test according to Equations (1) and (2) specified by ISO 13528 and EUPT. The deviations between the values obtained in this study were the thresholds. The permissible standard deviation of the test was calculated by setting it to 25% of the certified value (set value), which was established as a robust mean value for the institutions participating in the proficiency test.

s_s_^2^ = s_x_^2^ − (s_w_^2^/2) (1)

c = *F*_1_(0.3*σ*_all_) + *F*_2_S_w_^2^ (2)

s_s_: standard deviation between samples. s_x_: standard deviation of the sample mean. s_w_: standard deviation within the sample. *σ*_all_: allowed standard deviation of certified values; *F*_1_, *F*_2_: 1.88, 1.01.

For storage stability evaluation, three samples were randomly selected from the ten samples chosen for homogeneity evaluation and analyzed three times: at the beginning of the analysis procedure, two weeks after the beginning of the analysis, and one day after the end of the analysis. The analysis procedure was repeated twice for each sample. Storage stability was evaluated according to the ISO 13528 standard. The average value (x_1_) of the results obtained for the first stability test sample was calculated, and 25% of this value was set as the standard deviation (σ). The sample was considered stable if the absolute value of the difference between x_1_ and the analysis mean value (x_i_) of the results of stability tests 2 and 3 was less than 0.3 × σ.

### 2.4. Determination of the Assigned Values and Calculation of the Z-Scores

The analytical proficiency of the participating laboratories was evaluated using z-scores according to the ISO/IEC 17043 method. Their values were calculated via the following procedure. The assigned values ($x_{pt}$) for evaluation were derived based on the robust statistics of the results obtained for all institutions participating in the proficiency test ($x_{i}$)). The uncertainties of the assigned values were computed as follows: If the uncertainty of the certified value was lower than 0.3 × FFP–$\sigma_{pt}$ (FFP: fixed fit-for-purpose relative standard deviation), the uncertainty was considered negligible. Furthermore, standard deviation of the z-score, FFP–$\sigma_{pt}$, was set to 25% of $x_{pt}$. Calculating the z-scores of the participating laboratories via Equations (3) and (4) revealed whether the evaluation procedure satisfied the ISO/IEC 17043 criteria.

(3) $$u(x_{pt})=1.25\times \frac{s^{*}}{\sqrt{p}}$$

(4) $$z_{i=}\frac{x_{i}-x_{pt}}{FFP-\sigma_{pt}}$$

$$\left|z\right|\;\leq \;2\;\mathrm{acceptable}$$

$$2<\left|z\right|<3\;\mathrm{questionable}$$

$$\left|z\right|\geq 3\;\mathrm{unacceptable}$$

### 2.5. Category A/B and Triple-A Evaluation

Before calculating the z-scores, the evaluation method for the participating laboratories considered false positives and false negatives. A false positive is obtained when a pesticide that has not been added is detected at the levels above the corresponding MRL, whereas a false negative results when the analysis of an added pesticide results in a quantification amount below the MRL. In this study, false positives were assigned a z-score of 5, and false negatives were assigned a z-score of −5. Additionally, a triple-A method was employed to evaluate the analytical capabilities of the participating laboratories rather than the specific pesticide concentrations [21].

The traditional EUPT Category A and B evaluation method places laboratories in Category A if they are able to analyze more than 90% of the evaluated objects and if more than 90% of the analyzed items have z-scores of 2 or less. Otherwise, the laboratories are classified as Category B. However, this evaluation method does not consider z-score values, which limits its accuracy in assessing the laboratory proficiency. Consequently, the triple-A evaluation method proposed by Valverde et al. was used in this study, which added false positives to the existing category method based on the X (analytical range) and Y (z-score acceptability rate) parameters [21].

### 2.6. Using Z-Scores for Assessing the Multiresidue Analysis Capabilities of Participating Laboratories

The proficiency of the participating laboratories in multiresidue analysis was assessed by calculating z-scores during proficiency testing. Five evaluation methods were employed, including the *RSZ* and *SSZ* methods. The *RSZ* method does not account for the differences between z-scores, potentially allowing good results to compensate for poor ones.

(5) $$RSZ=\frac{\sum_{izi}}{\sqrt{n}}$$

The *SSZ* method is based on a chi-squared distribution. The computed *SSZ* values are evaluated as “Good” at a 68.3% probability, “Satisfactory” at a 95.5% probability, and “Questionable” at a 99.7% probability. *SSZ* is calculated using the sum of all squared z-scores. However, the *SSZ* value depends on percentiles, and is strongly influenced by the number of data points and outliers.

(6) $$SSZ=\sum_{i}z_{i}^{2}$$

To address these limitations, the *RLP* evaluation method divides the *SSZ* by the number of data points and takes the square root of the obtained value. RLP evaluation classifications include “Good (≤1.1),” “Satisfactory (>1.1, ≤1.35),” “Questionable (>1.1, ≤1.35),” and “Unsatisfactory (>1.6)”. However, the *RLP* technique has narrower classification intervals than those of the previously described z-score methods, which complicates the explanation of the differences between the results obtained near the threshold (7)

(7) $$RLP=\sqrt{\frac{SSZ}{n}}$$

To mitigate this issue, Medina-Pastor et al. [22] proposed the *SWZ* and *SZ*2 methods, based on the existing *SSZ* and *RSZ* techniques, which adjust the weights according to the z-score values, thereby reducing the effect of good z-scores and compensating for poor results. The *SWZ* method applies three categories of weights ($\omega \left(z_{i}\right)$) to individual z-score values (9). The *SWZ*-based evaluations are classified as “Good (≤2),” “Satisfactory (>2, ≤3),” and “Unsatisfactory (>3)”. However, this method may result in significant differences in values near the threshold owing to the assignment of different weights to the three categories.

(8) $$SWZ=\frac{\sum_{i=1}^{n}\left|z_{i}\right|\omega \left(z_{i}\right)}{n}$$

(9) $$\omega \left(z_{i}\right)=\left\{\begin{array}{c} 1=if\left|z\right|\leq 2\\ 3=if2<\left|z\right|\leq 3\\ 5=if\left|z\right|>3 \end{array}\right.$$

*SZ*2 is similar to *SWZ* (10); however, in this method, the weights $\omega \left(z_{i}\right)$ are not categorized and are equal to z-scores (11), thus addressing the *SWZ* issues. Hence, all methods must be compared to determine the most suitable approach.

(10) $$SZ2=\frac{\sum_{i=1}^{n}\left|z_{i}\right|\omega \left(z_{i}\right)}{n}$$

(11) $$\omega \left(z_{i}\right)=\left|z_{i}\right|$$

### 2.7. Submission of Analytical Information by the Participating Laboratories

The laboratories participating in the proficiency testing submitted their analytical results and provided additional information including the analyst experience, sample weights, pretreatment methods used, cleanup procedures, extraction solvents, extraction times, and calibration curve construction methods. However, factors that were difficult to classify or were applicable across all laboratories were excluded. Consequently, the following four factors were considered: analyst experience, presence or absence of a dispersive solid phase extraction (d-SPE) cleanup step, sample weight, and calibration curve construction method. The grouped analytical results were evaluated using the RLP, SMZ, and *SZ*2 methods. Statistical analysis was performed using the Mann–Whitney U test in Jamovi software (version 2.3.21). A statistical significance threshold was set to *p* ≤ 0.05.

## 3. Results and Discussion

### 3.1. Homogeneity and Stability of the Proficiency Test Results

The homogeneity characteristics of the proficiency test results obtained for the 10 sample bottles are presented in Table 1. The homogeneity and stability of the obtained numbers were evaluated according to the ISO 13528 standards. Cochran’s test was conducted to check for the presence of outliers, and all of the obtained results were below the established threshold value of 0.602, thus indicating their suitability. The squared value of the standard deviation (s_s_) calculated for the seven pesticide samples was zero and was smaller than 0.3σ, thus confirming that the proficiency test samples were suitable for homogeneity testing.

To evaluate the storage stability of the proficiency test samples, the results of three analyses obtained over five weeks were considered. All samples were stored at −20 °C until the time of analysis. The residual concentrations of the seven test pesticides were in the range of 0.126 to 0.644 mg/kg in the first round of testing and in the range of 0.117 to 0.628 mg/kg in the third round of testing. To assess their storage stability, the differences in residual concentrations between the second and third rounds were compared with the first-round residual concentrations. The obtained values were smaller than 0.3σ_pt,_ thus indicating that the storage stability of the analyzed samples during the proficiency testing period was sufficiently high (Table 2).

### 3.2. Z-Score Evaluation Data for the Participating Laboratories

The proficiency testing results were evaluated using the z-score method in accordance with the ISO/IEC 17043 standard. The assigned values for the test pesticides calculated from the results provided by the participating laboratories ranged from 0.049 to 0.239 mg/kg. When compared with the spiked concentrations in the range of 0.05–0.25 mg/kg, the ferimzone and imidacloprid amounts were 82–83% of the spiked concentrations (Table 3), whereas the remaining pesticides produced a match of more than 95% (Figure S1). Among the tested pesticides, ferimzone and imidacloprid exhibited low recovery rates at the participating laboratories, which could be attributed either to their degradation or to the less effective homogenization during the 8 h industrial mixing process as compared with the other pesticides.

The suitability rate of the participating laboratories for proficiency testing ranged from 79 to 97% (Table 3). The z-score evaluation results demonstrated the lowest suitability rate of 79% for ferimzone and the highest rates (97%) for hexaconazole, imidacloprid, and isoprothiolane. The low ferimzone suitability can be attributed to its LC–MS/MS chromatograms obtained in the E and Z forms with different peak sizes. The ferimzone concentrations corresponding to the two chromatograms were quantified separately and summed. However, laboratories with “Unsatisfactory” evaluations had performed the quantitative analysis after summing the area values of the two chromatograms and constructing the calibration curve. Consequently, the z-scores of the laboratories with the “Questionable” and “Unsatisfactory” ratings ranged from −5.0 to 2.1 and 5.0. The second-lowest group included difenoconazole and fenoxanil with suitability rates of 85% and 88%, respectively. No significant differences in terms of the analytical experience, sample preparation methods, or calibration curve construction were observed between the suitable and failed laboratories. Other factors, such as the state of the reference materials or analytical instruments used in the laboratories may have affected the results. Two laboratories received “Unsatisfactory” (z-score > 3) evaluations for fenoxanil because of false negatives. Although they used the QuEChERS preparation method and an LC–MS/MS analysis procedure (similar to other laboratories), the differences in their results can be attributed to various errors such as the excessive storage period of the reference materials used in the laboratories or incorrect retention times set for multiple reaction monitoring in analytical instruments.

A false-positive result was also obtained for pendimethalin in one laboratory that employed a matrix-matched calibration (MMC) procedure for the quantitative analysis. Consequently, this false positive was not due to matrix effects but rather owing to the contamination of the analytical instrument, leading to the misidentification of another chromatogram as a pesticide and its erroneous quantification.

### 3.3. Evaluation Methods Using A/B and Triple-A Categories

Various evaluation methods were applied to assess the proficiency test results. Using the Category A/B method currently employed by the European Union Reference Laboratory for proficiency testing, 29 laboratories (83%) were classified as Category A, whereas the remaining six laboratories were placed in Category B (Table 4). The Category B laboratories exceeded the z-score benchmark of ±2 for three or more of the seven pesticides tested (except for laboratory 6). Additionally, difenoconazole and ferimzone exceeded the acceptable ranges in four of the six laboratories. However, the category-based evaluation method has several limitations, as it does not consider the range of z-scores and false positives. In practice, the laboratories with suitable pesticide proportions of 89% and 10% during z-score evaluation were both placed in Category B.

The triple-A method, which overcomes these limitations, classifies all Category A laboratories as AAA. However, among the Category B laboratories, ABA, ABB, and CCA are the three possible evaluation outcomes. The laboratories rated as Category A generally exhibit excellent overall analytical capabilities, reducing the likelihood of receiving poor evaluations for false positives and achieving suitable judgments for more than 90% of the analyzed pesticides based on their z-scores. Nevertheless, although the triple-A method allows proficiency evaluation across 27 subdivided categories, it does not consider z-score values. Consequently, the evaluation procedure may be insufficient for the laboratories yielding z-scores close to the ±2 standard or near zero.

### 3.4. Comparison of the Evaluation Methods Based on the Z-Scores of Participating Laboratories

The proficiency test results obtained for the participating laboratories were compared using five evaluation methods. In this study, the number of laboratories rated “Good” by the five methods did not vary significantly and ranged from 23 to 26. Additionally, the number of institutions classified as “Unsatisfactory” ranged from 6 to 12 (Table 5).

The RSZ method produced the highest proportion of laboratories in the “Good” category because it compensated for the poor results with high z-scores. The number of laboratories included in the “Unsatisfactory” category was six, half of the number generated by the SSZ method. Notably, laboratories 8 and 28 were rated “Good” by the RSZ method, but received different assessments by the other methods (Table S1). In both laboratories, one out of the seven test pesticides exhibited a z-score of −5, while the remaining test pesticides had four or more z-scores within ±1, compensating for the unsatisfactorily evaluations. Both laboratories were rated as “Unsatisfactory” by the four other evaluation methods.

Although the results of the remaining four evaluation methods (excluding RSZ) demonstrated a similar pattern, laboratories 14 and 32 produced different results (Table S1). Laboratory 14 was evaluated as “Unsatisfactory” by the SSZ and SZ2 methods, while laboratory 32 was rated “Unsatisfactory” by SSZ and “Good” by the other techniques. This occurred because the SSZ method used a relative evaluation criterion based on the chi-square distribution, which is significantly influenced by the number of participating laboratories and outliers, resulting in a conservative assessment during proficiency testing [22]. Moreover, SZ2 can generate “Poor” evaluations when many pesticides have z-scores close to the suitability criteria (±2), even if all the analyzed pesticide z-scores are suitable. Laboratory 14 was classified as “Questionable” with a z-score of −2.4 for ferimzone, and the remaining six pesticides exhibited z-scores ranging from −0.6 to 0.2 (close to 0); however, the same laboratory was rated “Unsatisfactory” by SZ2. Laboratory 32 generated suitable z-scores for all pesticides; however, the RSZ (−2.1), SSZ (8.7), and RLP (1.1) values were close to the “Unsatisfactory” values obtained for laboratory 14 (−1.3, 6.9, and 1.0, respectively), while SWZ and SZ2 produced lower values for laboratory 32, which were close to the “good” evaluation.

Laboratories 11, 13, 28, and 39, which had been classified as Category A using the category-based evaluation method, were rated “Unsatisfactory” by the other techniques except for RSZ (Table S2). These four laboratories included one pesticide with a z-score of +5 or −5 among the analyzed pesticides. The proposed methods were applicable for evaluating the proficiency of laboratories in the current multiresidue analysis system. In particular, the SZ2 and SWZ methods, which use correction factors based on z-score values, were found to be the most suitable.

However, a certain pattern was observed for placing the laboratories into the “Good” and “Unsatisfactory” categories by the four evaluation methods, excluding RSZ. As the actual proficiency of residual analysis laboratories is diverse, their evaluation classification must be diversified by further refining correction factors and evaluation calculation methods in the future.

### 3.5. Comparison of the Evaluation Results Based on Critical Analytical Conditions

**Figure 1:**
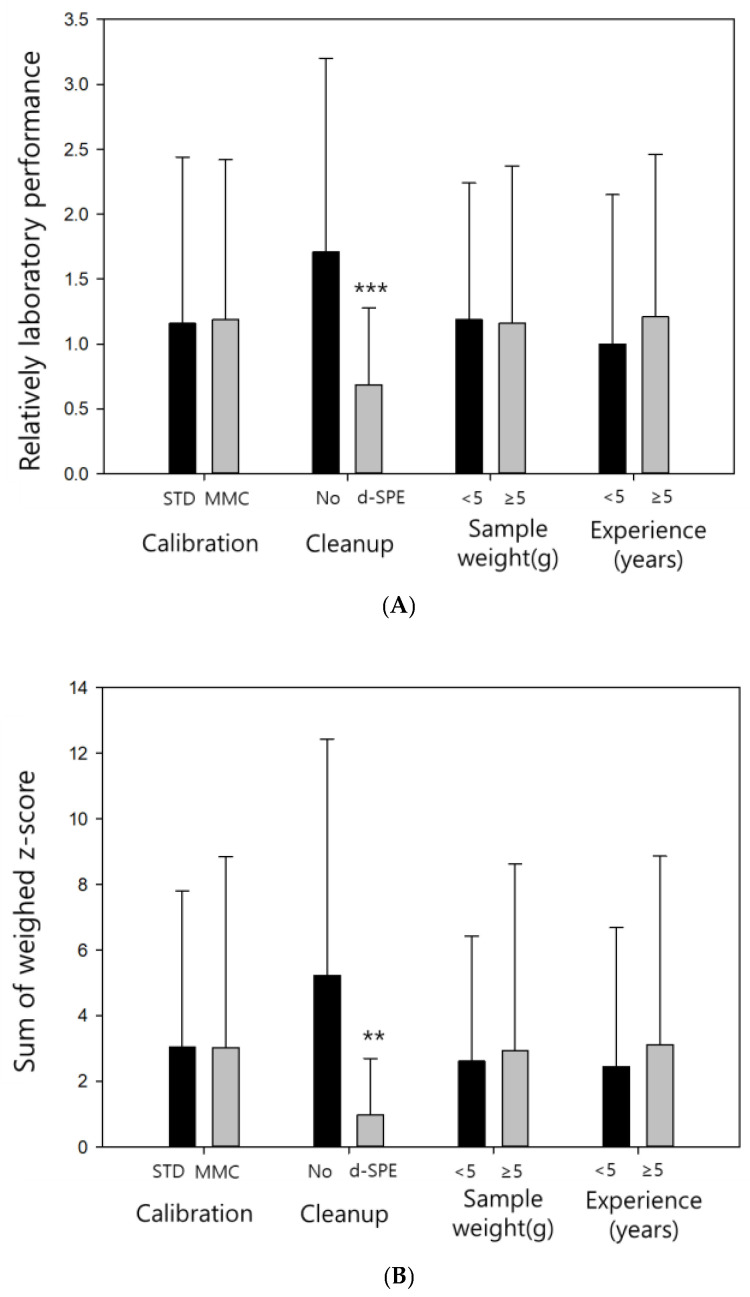

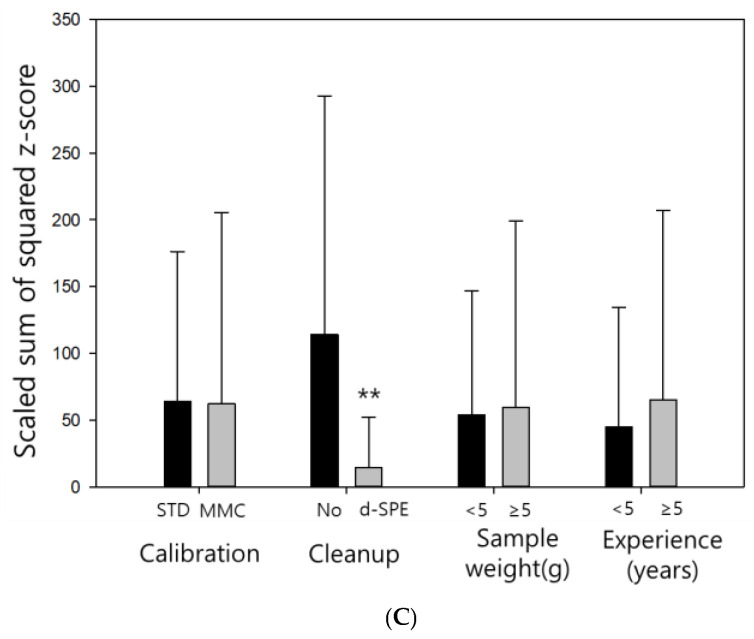
Evaluation results obtained using the (**A**) relatively laboratory performance(RLP); (**B**) sum of weighted z-score (SWZ); and (**C**) scaled sum of squared z-score (*SZ*2) pretreatment methods.

Basic analytical information was collected to compare the proficiency evaluation results obtained under various conditions and provide feedback for future analysts. The effects of four factors (calibration curve construction, sample cleanup procedure, sample weight, and analytical experience) on the results obtained by the RLP, SWZ, and SZ2 methods were investigated. Similar trends were observed for three of these factors (calibration curve construction, sample weight, and analytical experience) across all evaluation methods (Figure 1).

No significant differences in the evaluation results were obtained for the calibration curves constructed using the standard solution calibration (STD) and MMC techniques (*p* > 0.05). The samples studied in the proficiency test contained rice, which exhibited weak matrix effects during the multiresidue pesticide analysis via QuEChERS [29]; therefore, the results obtained using STD and MMC were almost identical. However, for the samples with high impurity contents, the differences between STD and MMC caused by the matrix effects can exceed ±50%, potentially affecting the analysis results. Therefore, the use of MMC is recommended in the present study.

No significant differences between the obtained results were caused by sample weight variations. The use of an appropriate sample size is important for obtaining accurate values. However, when samples are homogenized with dry ice and subjected to a suitable grinding process [30], the obtained data should not vary due to different sample weights. Furthermore, in the proficiency tests, samples were shipped after achieving homogeneity, and consequently differences caused by sample weight were not observed in this study.

Although the differences in evaluation results owing to analytical experience were not significantly different (*p* > 0.05), the average evaluation data were lower for the analysts with less than five years of experience. Recently, QuEChERS has been widely used in pesticide residue analysis because it is relatively simple and produces reliable results, making it suitable for beginners [31]. When a QuEChERS-based preprocessing step is performed, analytical experience is not expected to be a decisive factor affecting the results.

Better evaluation results were obtained for the laboratories that performed a d-SPE cleanup process (** *p* < 0.01). According to the evaluation data, the RLP method produced significantly different data with *p*-values of 0.001 (***) or lower, depending on the cleanup procedure, whereas the other two evaluation methods resulted in *p*-values of 0.01 or lower. In the multiresidue analysis performed using QuEChERS, cleanup is an important factor for obtaining good results. Since the analysis efficiency increases with an increase in the cleanup efficiency, the laboratories that perform the d-SPE cleanup procedure should produce better evaluation data [32].

In the multiresidue analysis of rice using QuEChERS, d-SPE cleanup was essential for obtaining good evaluation results. Implementing the cleanup process could also be an important feedback point for improving the proficiency of residual pesticide analysis laboratories in the future.

## 4. Conclusions

The homogeneity and storage stability of the proficiency test samples were sufficiently high according to the ISO 13528 standard. The participating laboratories were evaluated for each pesticide using z-scores according to the ISO/IEC 17043 standard with suitability rates ranging from 79% to 97%. The low suitability rates obtained for ferimzone and imidacloprid were due to either their decomposition during the homogenization process or their low recovery rates in the analytical methods used by the analysts. The evaluation methods used by the participating laboratories were the category A/B and triple-A categories. The Category A/B method did not consider the range and aspect proportion of z-scores, whereas the triple-A method enabled a more detailed evaluation procedure but did not consider the z-score values. Therefore, both methods require significant improvements, and new evaluation methods that can further differentiate between the capabilities of different laboratories must be developed. Various methods for evaluating the proficiency test results obtained by the laboratories participating in the residual pesticide analysis were compared. The results revealed that the *SZ*2 and *SWZ* methods were the most suitable for evaluating the proficiency of laboratories for multiresidue analysis. However, the current evaluation methods exhibit a pattern primarily consisting of the “Good” and “Unsatisfactory” groups. In the future, diversifying the evaluation classification system by improving the correction coefficients and evaluation calculation methods used will be necessary. By implementing this approach, various laboratory proficiencies can be evaluated more accurately. Additionally, this study investigated the effects of four major analytical parameters (calibration curve, sample cleanup, sample weight, and analytical experience) on the proficiency evaluation results obtained by the *RLP*, *SWZ*, and *SZ*2 methods. For the calibration curve construction, sample weight, and analytical experience, no significant differences were observed between two different groups of data analyzed by the three evaluation techniques (*p* > 0.05). However, the evaluation results submitted by the institutions that performed the cleanup procedure were better than those without a cleanup (*p* < 0.01), thus confirming that cleanup plays an important role in improving the reliability of the multiresidue analysis results produced using QuEChERS.

## Figures and Tables

**Table 1:** Homogeneity test results obtained for seven pesticides in the studied rice samples during proficiency testing.

Compound	Homogeneity Test					Cochran’s Test		
	σ_pt_	σ^2^_all_	S_sample_^2^	c	S_sample_^2^ < c	Cochran	CV ^1)^	C < CV
Chlorantraniliprole	0.031	0.00009	0.000000	0.0003	Accept	0.376	0.602	Accept
Difenoconazole	0.053	0.00025	0.000000	0.00203	Accept	0.290	0.602	Accept
Fenoxanil	0.009	0.000007	0.000000	0.00011	Accept	0.593	0.602	Accept
Ferimzone	0.034	0.00010	0.000000	0.0005	Accept	0.373	0.680	Accept
Hexaconazole	0.017	0.00002	0.000000	0.00019	Accept	0.456	0.602	Accept
Imidacloprid	0.169	0.00256	0.000000	0.0079	Accept	0.378	0.602	Accept
Isoprothiolane	0.070	0.00044	0.000000	0.0014	Accept	0.296	0.638	Accept

^1)^ Coefficient of Variation.

**Table 2:** Storage stability of the seven pesticide samples evaluated during proficiency testing.

Compound	1st	2nd	3rd	0.3σ_pt_	Stability Test	
					(1st–2nd) < 0.3σpt	(1st–3rd) < 0.3σpt
Chlorantraniliprole	0.126	0.125	0.117	0.009	passed	passed
Difenoconazole	0.254	0.237	0.242	0.019	passed	passed
Fenoxanil	0.031	0.03	0.024	0.002	passed	passed
Ferimzone	0.150	0.144	0.139	0.011	passed	passed
Hexaconazole	0.072	0.068	0.068	0.005	passed	passed
Imidacloprid	0.644	0.619	0.628	0.048	passed	passed
Isoprothiolane	0.361	0.34	0.309	0.027	passed	passed

**Table 3:** Assigned values and z-scores determined for the test pesticides.

Compound	Assigned Value (mg/kg)	Spiked Concentration (mg/kg)	σ_pt_	Z-Score		
				Acceptable (%)	Questionable (%)	Unacceptable (%)
Chlorantraniliprole	0.239	0.250	0.060	94 (31)	0 (0)	6 (2)
Difenoconazole	0.079	0.080	0.020	85 (28)	0 (0)	15 (5)
Fenoxanil	0.060	0.060	0.015	88 (28)	0 (0)	13 (4)
Ferimzone	0.083	0.100	0.021	79 (27)	6 (2)	15 (5)
Hexaconazole	0.086	0.090	0.021	97 (33)	0 (0)	3 (1)
Imidacloprid	0.049	0.060	0.012	97 (32)	0 (0)	3 (1)
Isoprothiolane	0.060	0.060	0.015	97 (32)	0 (0)	3 (1)

**Table 4:** Evaluation data classified using the A/B and triple-A categories.

Evaluation	Classification			
Category	A	B		
	29	6		
Triple-A	AAA	ABA	ABC	CCA
	29	4	1	1

**Table 5:** Proficiency evaluation results obtained for the participating laboratories by different methods.

Interpretation	RSZ	SSZ	RLP	SWZ	SZ2
Good	26 (74%)	23 (66%)	25 (71%)	25 (71%)	24 (69%)
Satisfactory	–	–	–	–	–
Questionable *	3 (9%)	N.S. **	–	N.S.	N.S.
Unsatisfactory	6 (17%)	12 (34%)	10 (29%)	10 (29%)	11 (31%)

* RLP is included in the interpretation, and both the low and high standards are considered “Questionable” in RSZ. ** No standard.

## Data Availability

Data is contained within the article or Supplementary Material.

## Supplementary Materials

The following supporting information can be downloaded at: https://www.mdpi.com/article/10.3390/foods12102085/s1, Figure S1: Evaluation results of pesticide-specific z-score values for the participating laboratories, Table S1: Comparison of the z-scores of the tested pesticides in laboratories with different evaluation results, Table S2: Differences in the laboratory results between the Category A, B and Triple-A evaluation methods and the z-score-based evaluation methods.
