# Supplementary material for: Korean Proficiency Tests for Pesticide Residues in Rice: Comparison of Various Proficiency Testing Evaluation Methods and Identification of Critical Factors for Multiresidue Analysis
Source: Foods. 2023 May 22;12(10):2085. doi: 10.3390/foods12102085 (PMC10217400; doi:10.3390/foods12102085)
Supplement: Supplementary file 1 [file foods-12-02085-s001.zip › foods-2407004-supplementary.pdf]

SUPPLEMENTARY MATERIAL

Korean proficiency test for pesticide residue in rice: Comparison of various proficiency test evaluation methods and identification of critical factors for multi-residue analysis

Hyosub Lee [1*], Gunhee Jung [1], Juhyeon Min [2], Hyanghee Kim [1], Wontae Jeong [1] and Taekkyum Kim [1]

[1] **National Institute of Agricultural Sciences, 166 Nongsaengmyeong-ro, Iseo-myeon, Wanju-gun, Jellabuk-do, 55365, Korea**

[2] **Department of Food Science and Technology, Kyungpook National University, 25, Sangyeok-ro 14-gil, Buk-gu, Daegu, 41536, Korea**

*Corresponding author. Fax: +82 63 238 3238

Email address: lhs8255@korea.kr

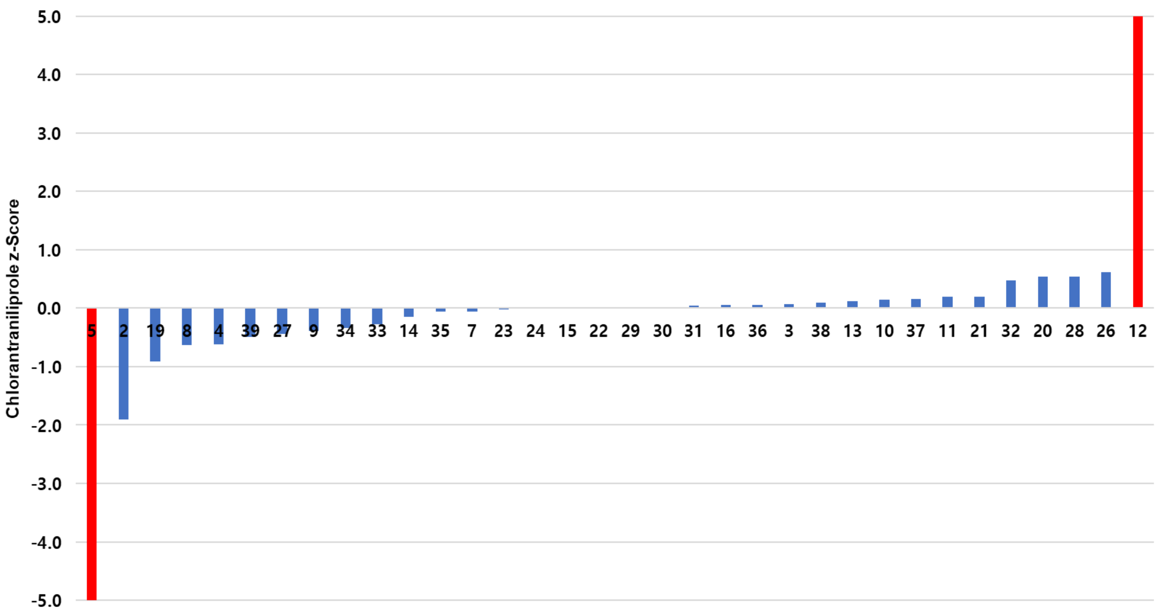
Chloranttaniliprole z-Score
5.0
4.0
3.0
2.0
1.0
0.0
-1.0
-2.0
-3.0
-4.0
-5.0
5 2 19 8 4 39 27 9 34 33 14 35 7 23 24 15 22 29 30 31 16 36 3 38 13 10 37 11 21 32 20 28 26 12

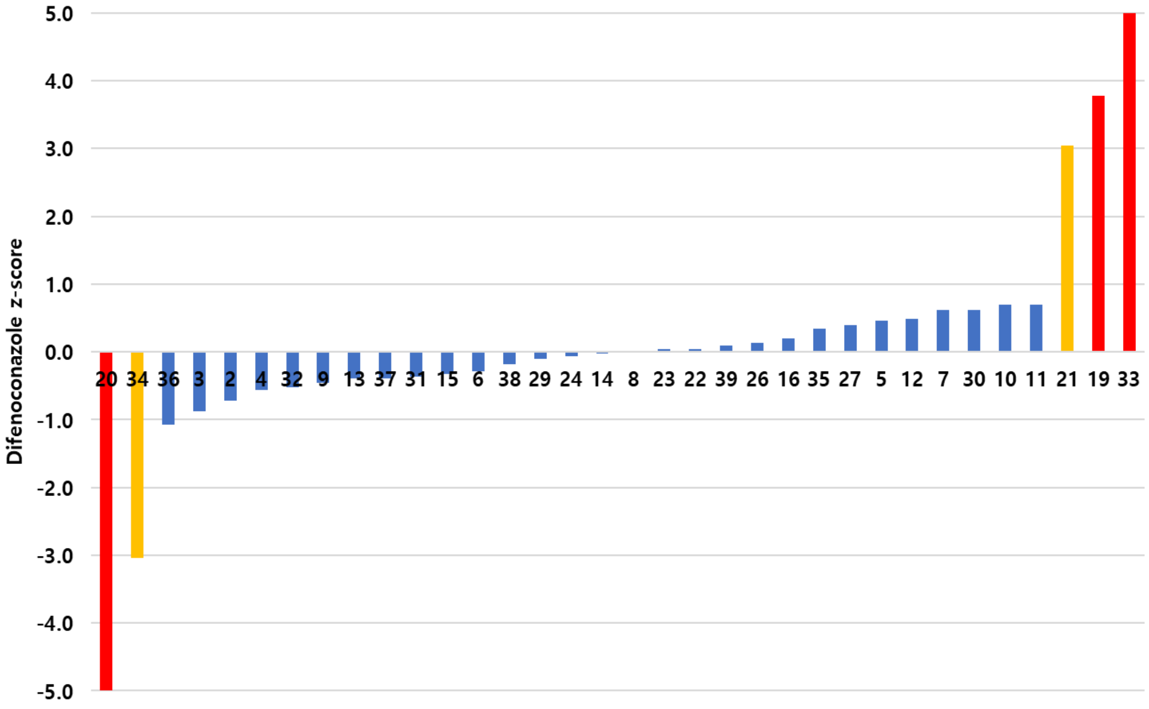
Difenoconazole z-score
5.0
4.0
3.0
2.0
1.0
0.0
-1.0
-2.0
-3.0
-4.0
-5.0
20 34 36 3 2 4 32 9 13 37 31 15 6 38 29 24 14 8 23 22 39 26 16 35 27 5 12 7 30 10 11 21 19 33

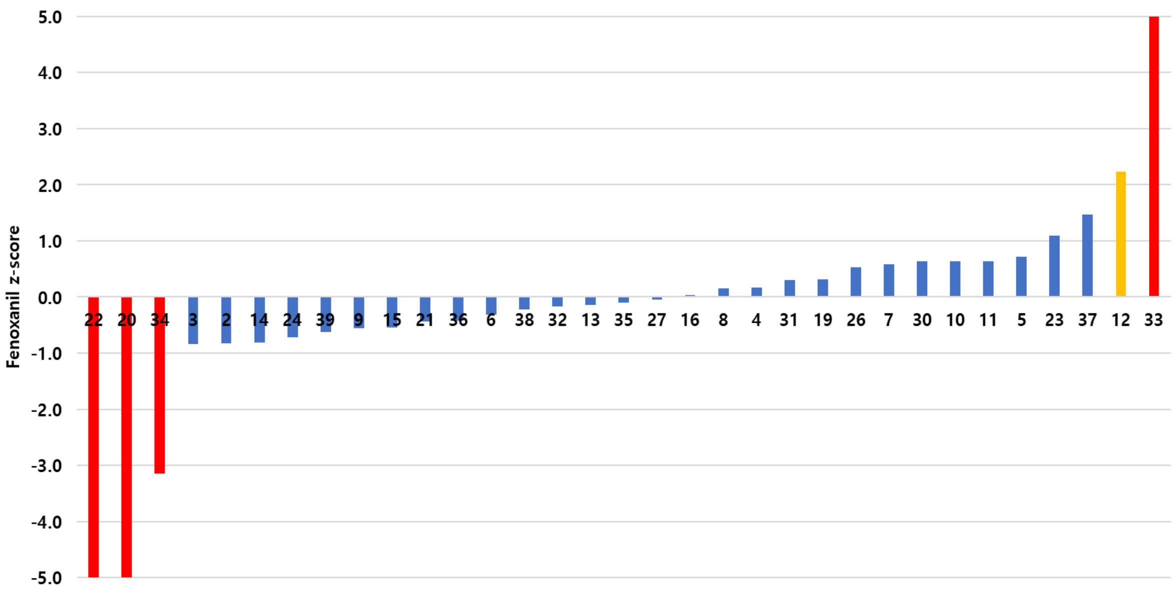
Fenoxanil z-score
5.0
4.0
3.0
2.0
1.0
0.0
-1.0
-2.0
-3.0
-4.0
-5.0
22 20 34 3 2 14 24 39 9 15 21 36 6 38 32 13 35 27 16 8 4 31 19 26 7 30 10 11 5 23 37 12 33

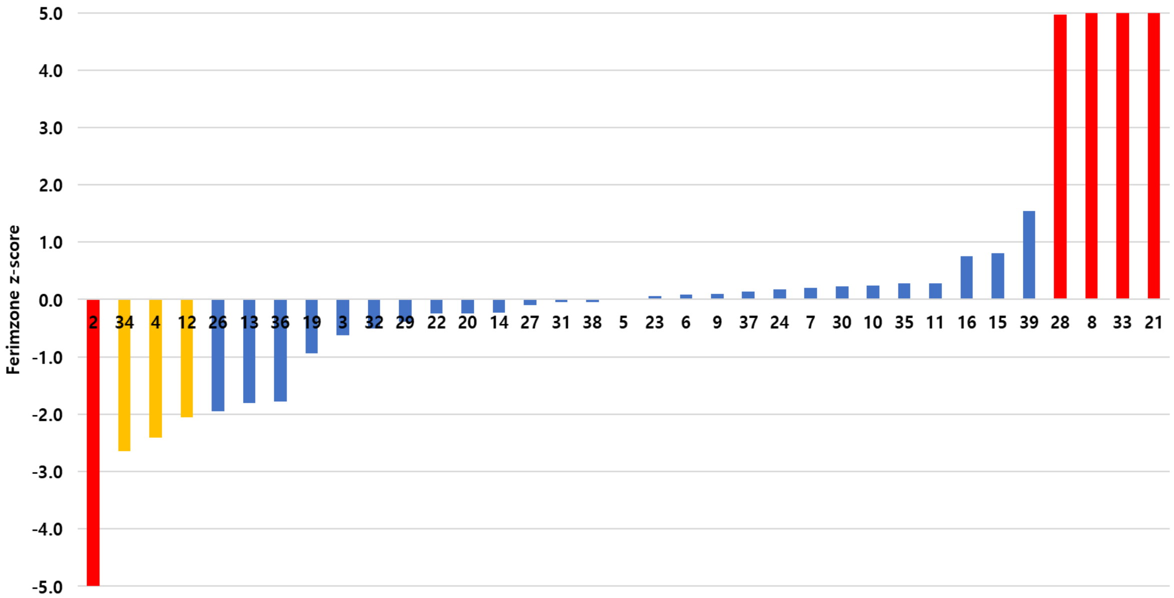
Ferimzone z-score
5.0
4.0
3.0
2.0
1.0
0.0
-1.0
-2.0
-3.0
-4.0
-5.0
2 34 4 12 26 13 36 19 3 32 29 22 20 14 27 31 38 5 23 6 9 37 24 7 30 10 35 11 16 15 39 28 8 33 21

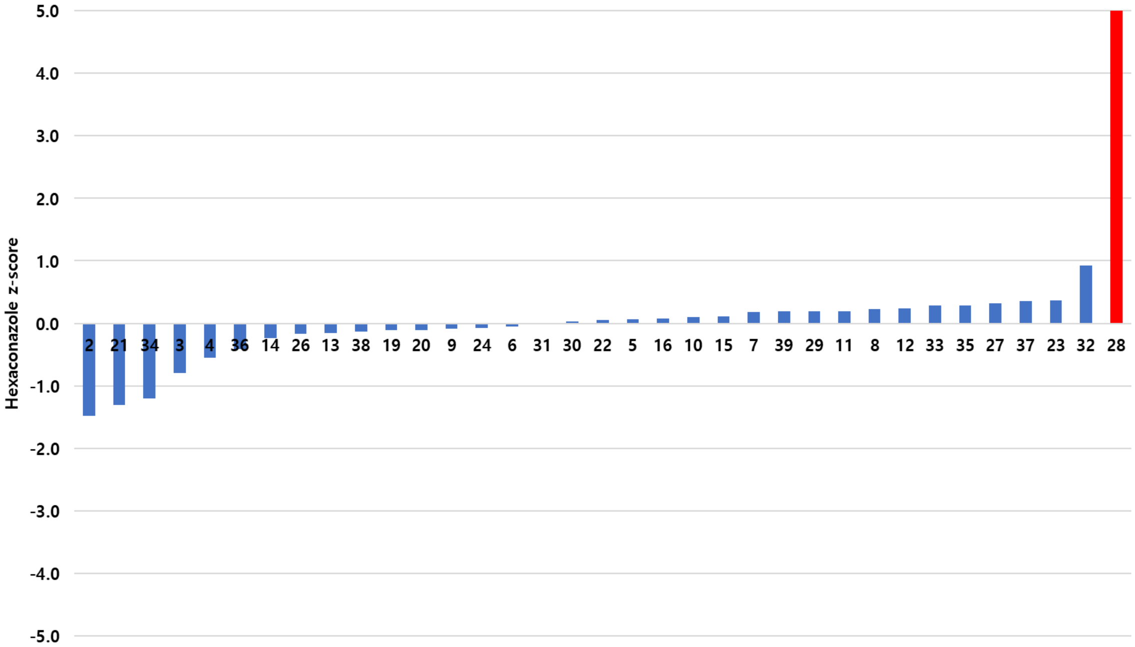
Hexaconazole z-score
5.0
4.0
3.0
2.0
1.0
0.0
-1.0
-2.0
-3.0
-4.0
-5.0
2 21 34 3 4 36 14 26 13 38 19 20 9 24 6 31 30 22 5 16 10 15 7 39 29 11 8 12 33 35 27 37 23 32 28

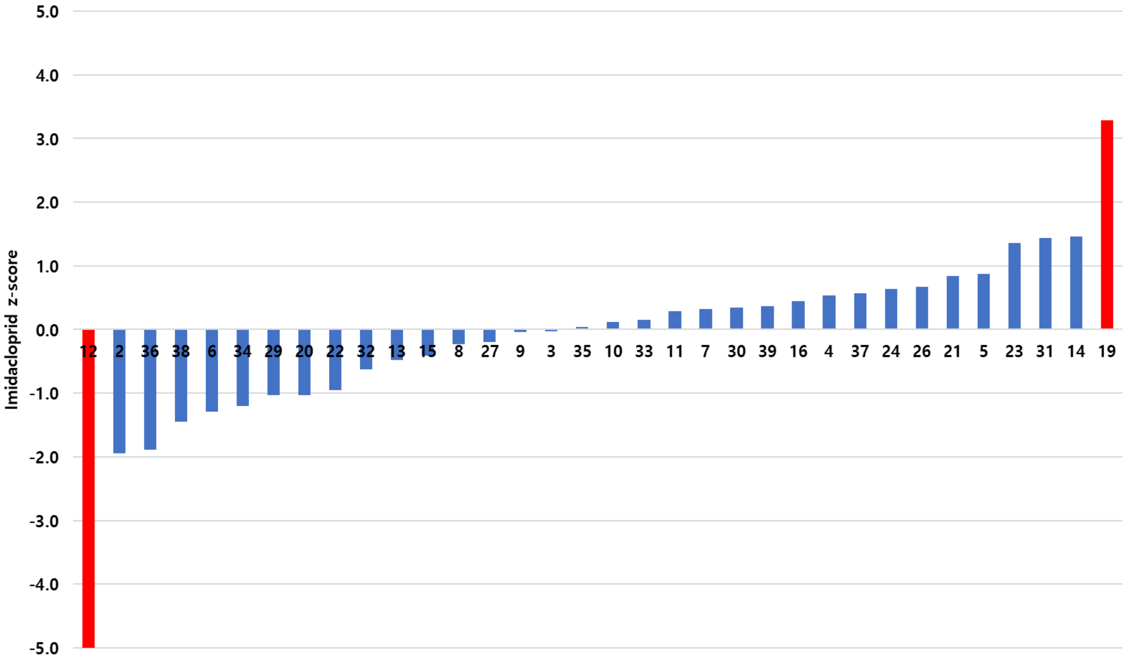
Imidacloprid z-score
5.0
4.0
3.0
2.0
1.0
0.0
-1.0
-2.0
-3.0
-4.0
-5.0
12 2 36 38 6 34 29 20 22 32 13 15 8 27 9 3 35 10 33 11 7 30 39 16 4 37 24 26 21 5 23 31 14 19

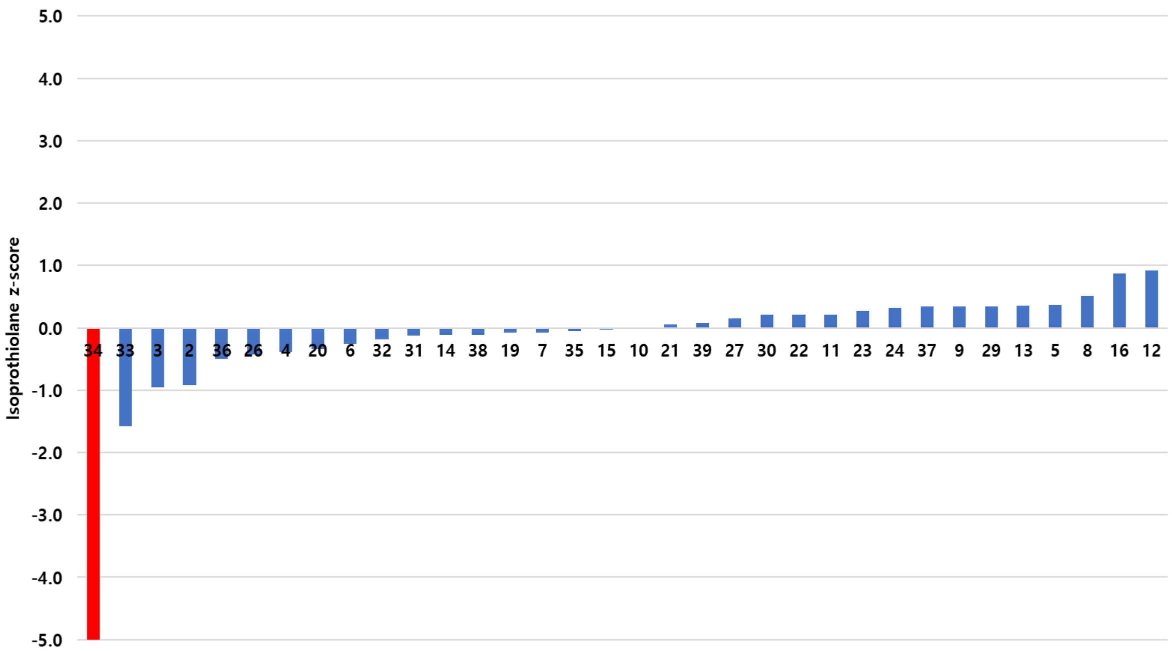

Figure S1. Evaluation results of pesticide-specific z-score values for the participating laboratories

Table S1. Comparison of the z-scores of the tested pesticides in laboratories with different evaluation results

Lab No.	Chlorantraniliprole	Difenoconazole	Fenoxanil	Ferimzone	Hexaconazole	Imidacloprid	Isoprothiolane	RSZ	SSZ	RLP	SWZ	SZ2
8	-0.63	0.49	2.24	-2.06	0.24	-5.00	0.91	-1.4	35.8	2.3	5.7	101.4
28	0.54	0.04	-5.00	-0.24	0.05	-0.95	0.21	-2.0	26.3	1.9	3.9	89.5
14	-0.14	-0.56	0.17	-2.41	-0.56	0.53	-0.39	-1.3	6.9	1.0	1.4	7.6
32	0.48	-1.07	-0.43	-1.78	-0.42	-1.89	-0.50	-2.1	8.7	1.1	0.9	1.2

Table S2. Differences in the laboratory results between the Category A, B and Triple-A evaluation methods and the z-score-based evaluation methods

Lab No.	Category	X	Y	Z	RSZ	SSZ	RLP	SWZ	SZ2
11	A	A	A	A	-4.0	33.1	2.2	4.4	90.4
13	A	A	A	A	2.2	25.4	1.9	3.8	89.3
28	A	A	A	A	-2.0	26.3	1.9	3.9	89.5
39	A	A	A	A	2.5	37.2	2.3	6.2	122.9
